# Supplementary material for: Harbouring public good mutants within a pathogen population can increase both fitness and virulence
Source: eLife. 2016 Dec 28;5:e18678. doi: 10.7554/eLife.18678 (PMC5193496; doi:10.7554/eLife.18678)
Supplement: Figure 3—source data 1. — DOI: http://dx.doi.org/10.7554/eLife.18678.013 [file elife-18678-fig3-data1.docx]

| 1. Sucrose | | | | | |
| --- | --- | --- | --- | --- | --- |
| parameter | estimate | standard error estimate | T value | p value |  |
| $c_{hi}$ | 3.7058 x 10^-5^ | 8.597 x 10^-24^ | 4.3106 x 10^18^ | < 10^-15^ |  |
| $p$ | 1 x 10^10^ | 8.9348 x 10^-31^ | 1.1192 x 10^40^ | < 10^-15^ |  |
| $c_{lo}$ | 6.6768 x 10^-14^ | 8.0478 x 10^-15^ | 8.2967 | < 10^-7^ |  |
| $V_{max}$ | 9.8624 x 10^12^ | 1.1727 x 10^-16^ | 8.4101 x 10^28^ | < 10^-15^ |  |
| $K_{m}$ | 0.12247 | 0.0045833 | 26.721 | < 10^-15^ |  |
| 1. Glucose | | | | |  |
| parameter | estimate | standard error estimate | T value | p value |  |
| $c_{hi}$ | 2.3203 x 10^-5^ | 4.0433 x 10^-24^ | 5.7385 x 10^18^ | < 10^-15^ |  |
| $p$ | 1 x 10^10^ | 4.2022 x 10^-31^ | 2.3797 x 10^40^ | < 10^-15^ |  |
| $c_{lo}$ | 2.8892 x 10^-14^ | 3.785 x 10^-15^ | 7.6331 | < 10^-7^ |  |
| $V_{max}$ | 1.8711 x 10^13^ | 6.072 x 10^-17^ | 3.0816 x 10^29^ | < 10^-15^ |  |
| $K_{m}$ | 0.1499 | 0.0039922 | 37.547 | < 10^-15^ |  |

**Figure 3 – source data 1:** Typical parameter estimates obtained by fitting the geometric form of the rate-efficiency trade-off ^38^ to data in Figure 3 of the main text.
