# Supplementary material for: Harbouring public good mutants within a pathogen population can increase both fitness and virulence
Source: eLife. 2016 Dec 28;5:e18678. doi: 10.7554/eLife.18678 (PMC5193496; doi:10.7554/eLife.18678)
Supplement: Supplementary file 1. — DOI: http://dx.doi.org/10.7554/eLife.18678.016 [file elife-18678-supp1.docx]

**Supplementary file 1 for ‘Harbouring public good mutants within a pathogen population can increase both fitness and virulence’**

Richard J. Lindsay, Michael J. Kershaw, Bogna J. Pawlowska, Nicholas J. Talbot and Ivana Gudelj

School of Biosciences, University of Exeter, Geoffrey Pope Building, Stocker Road, Exeter, EX4 4QD, UK.

| **Supplementary File 1: Primers used in this study** | |
| --- | --- |
| Primer name | Nucleotide sequence (5’-3’) |
| INV1_50.1 | TTGGCGTGGAGAAAGAAGTGGGTA |
| INV1_M13F | GTCGTGACTGGGAAAACCCTGGCGGTGCTGGTATTATTAACGAGGGAC |
| INV1_30.1 | AAAGATCAAGGACAAGGCACCCAA |
| INV1_M13R | TCCTGTGTGAAATTGTTATCCGCTGGGCGGTCATGTTTGAGAGATAGG |
| IL_M13F | CGCCAGGGTTTTCCCAGTCACGAC |
| ILsplit | TCTGGTTGTATTCTCAGGAC |
| LVsplit | CATACCAAGCATGTGCAGTG |
| LV_M13R | AGCGGATAACAATTTCACACAGGA |
| INV1_comp_F_EcoRi | GAATTCTTCAGATTATGTGTATGGCGGC |
| INV1_comp_R_Hindiii | AAGCTTATATGTCGGCTGTCTTTCTCCA |
| BAR_F_Noti | GCGGCCGCAAGTCGACAGAAGATGATATTGAAGG |
| BAR_R_Spei | ACTAGTAAGTCGACCTAAATCTCGGTGA |
| ToxA_F_inBAR | CTACACCCACCTGCTGAAGT |
| ToxA_R_NcoI | CCATGGAAGGACTATATTCATTCAATGTCAGC |
| trpC_F_NdeI | CATATGAAAGCGGCCGCCCGGCTGCAGC |
| trpC_R_xhoi | CTCGAGTGGAGATGTGGAGT |
| GPDp_F_Hindiii  GPDp_R_EcoRi  INV1_ORF_F  INV1_ORF_R | AAGCTTTTACTTTGTAAAACGACGGCCA  GAATTCAAGACTAACTATAAAAGTAGAA  TTAAGCTTATGAAATTCACATTTGTGTCATCG  AAGAATTCTTACCATCCGTTCCACCAGGTGTA |
