# Supplementary material for: Harbouring public good mutants within a pathogen population can increase both fitness and virulence
Source: eLife. 2016 Dec 28;5:e18678. doi: 10.7554/eLife.18678 (PMC5193496; doi:10.7554/eLife.18678)
Supplement: Supplementary file 2. — DOI: http://dx.doi.org/10.7554/eLife.18678.017 [file elife-18678-supp2.pdf]

Supplementary file 2 - Infection assay protocol - 'Harbouring public good mutants within a pathogen population can increase both fitness and virulence'

Richard J. Lindsay, Michael J. Kershaw, Bogna J. Pawlowska, Nicholas J. Talbot and Ivana Gudelj
School of Biosciences, University of Exeter, Geoffrey Pope Building, Stocker Road, Exeter, EX4 4QD, UK.

1) 21-day old rice seedlings (cultivar CO39) were used for Rice Blast infections. 6-8 plants grew from each pot. Each pot was randomly allocated to an infection treatment (e.g. 20% Guy11 + 80% Δ*inv1*). Plants were grown, and infections took place, in a controlled environment for humidity (70-82%), aeration, temperature (24 °C) and light regime (12 h dark/light cycle) and intensity.

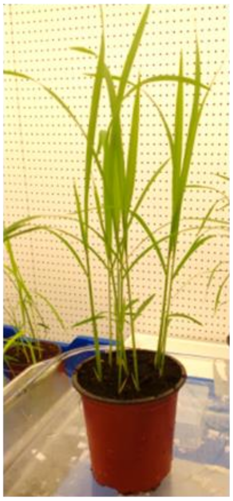

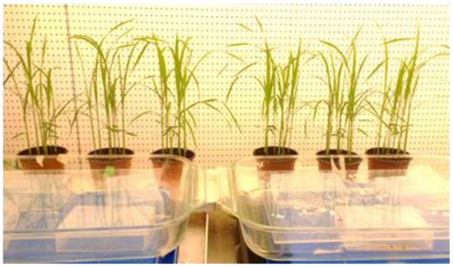

2) Randomly allocated pots of rice plants were positioned in random order side by side where they experienced the same growth environment and conditions within the rice growth room.

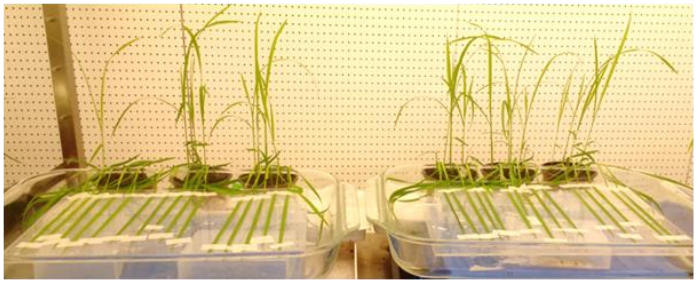

3) A single leaf per plant was used for infection by spot inoculation. Attached leaves were affixed to a plastic sheet to facilitate inoculation. These were secured within a basin so that a high humidity environment could be generated to facilitate successful infection

4) Spore suspensions ($5 \times 10^{4}.ml^{-1}$) were inoculated onto leaves with 20 µl droplets

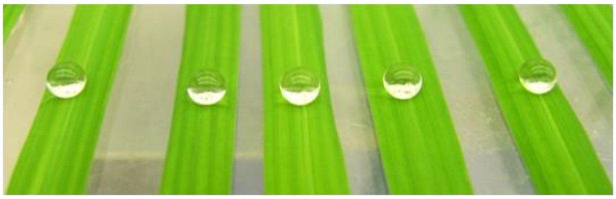

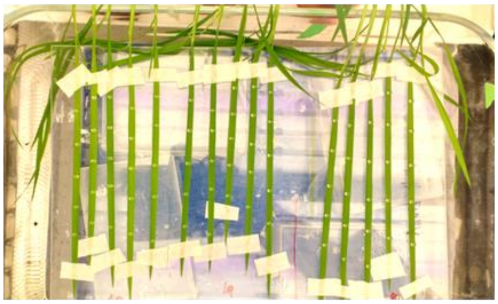

5) Inoculated leaves were covered with cling film to create the high humidity conditions to facilitate successful infection. This also ensured constant conditions for all infections. Basins were filled with approximately 500 ml of water to maintain saturating humidity.

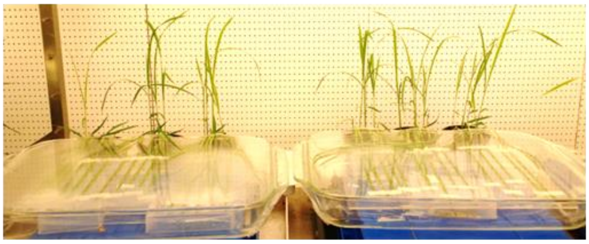

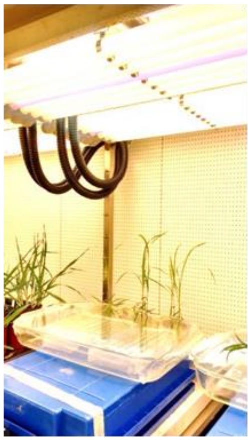

6) Infections were allowed to proceed for 7 d under normal growth conditions. After 7 d, disease patches were excised and placed under high humidity to induce conidiation. Conidia were quantified for 14 lesions per treatment after a further 2 d.
